# CB2 Cannabinoid Receptor Knockout in Mice Impairs Contextual Long-Term Memory and Enhances Spatial Working Memory

**DOI:** 10.1155/2016/9817089

**Published:** 2015-12-27

**Authors:** Yong Li, Jimok Kim

**Affiliations:** ^1^Department of Neuroscience and Regenerative Medicine, Medical College of Georgia, Georgia Regents University, Augusta, GA 30912, USA; ^2^Department of Neurology, Medical College of Georgia, Georgia Regents University, Augusta, GA 30912, USA

## Abstract

Neurocognitive effects of cannabinoids have been extensively studied with a focus on CB1 cannabinoid receptors because CB1 receptors have been considered the major cannabinoid receptor in the nervous system. However, recent discoveries of CB2 cannabinoid receptors in the brain demand accurate determination of whether and how CB2 receptors are involved in the cognitive effects of cannabinoids. CB2 cannabinoid receptors are primarily involved in immune functions, but also implicated in psychiatric disorders such as schizophrenia and depression. Here, we examined the effects of CB2 receptor knockout in mice on memory to determine the roles of CB2 receptors in modulating cognitive function. Behavioral assays revealed that hippocampus-dependent, long-term contextual fear memory was impaired whereas hippocampus-independent, cued fear memory was normal in CB2 receptor knockout mice. These mice also displayed enhanced spatial working memory when tested in a Y-maze. Motor activity and anxiety of CB2 receptor knockout mice were intact when assessed in an open field arena and an elevated zero maze. In contrast to the knockout of CB2 receptors, acute blockade of CB2 receptors by AM603 in C57BL/6J mice had no effect on memory, motor activity, or anxiety. Our results suggest that CB2 cannabinoid receptors play diverse roles in regulating memory depending on memory types and/or brain areas.

## 1. Introduction

Neuropsychiatric effects of cannabinoids, including endocannabinoids and cannabis ingredients, have been primarily studied in relation to CB1 cannabinoid receptors (CB1Rs) because CB1R has been considered the major, if not the only, cannabinoid receptor in the nervous system. Although early studies showed that CB2 cannabinoid receptors (CB2Rs) are expressed only in the immune system but not in the brain [1–3], recent evidence has indicated that CB2Rs are also present in the brain (for review, see [4]).

In situ hybridization studies show that CB2R mRNAs are expressed in neurons in the cerebellum [5], globus pallidus, cerebral cortex, hippocampus [6, 7], ventral tegmental area [8], nucleus accumbens, and dorsal striatum [9] in rodents and macaque. These data have been supported by negative control experiments with CB2R knockout (KO) mice [8] or sense probes [5, 7, 8]. The detection of CB2R proteins using anti-CB2R antibodies has been controversial [10–13] perhaps because of the low expression levels of CB2Rs and/or poor specificity of the currently available antibodies. The expression of CB2Rs in microglia can be induced under pathological conditions for neuroprotective immune responses (for review, see [14]).

CB1Rs are unequivocally involved in many neurocognitive effects induced by cannabinoids (for review, see [15]), but it is unclear whether CB2Rs also participate in neurological effects. Δ^9^-Tetrahydrocannabinol (THC), the primary psychoactive component of marijuana, binds to CB1R and CB2R with the same affinity [16]. Anandamide and 2-arachidonoylglycerol, two main endocannabinoids, can also activate both CB1R and CB2R with a 3- to 4-fold higher affinity for CB1R than for CB2R although anandamide and Δ^9^-THC are low-efficacy agonists of CB2Rs [16–18]. Therefore, it is conceivable that both receptors in the brain might be activated when levels of endocannabinoids are elevated or after long-term intake of marijuana.

Evidence suggests that CB2Rs modulate neuronal functions. Activation of CB2Rs reduces pain (for review, see [19]), impulsive behaviors [20], and locomotor activity [21–23] of rodents and also vomiting of ferrets [24]. Chronic activation or blockade of CB2Rs in rodents increases or decreases, respectively, anxiety [25]. Activation of CB2Rs decreases the excitability of peripheral sensory neurons [19], cortical pyramidal neurons [26], and dopaminergic neurons in the ventral tegmental area [8]. CB2Rs modulate excitatory synapses in the hippocampus [27, 28] as well as inhibitory synaptic transmission [25, 29, 30].

In humans, the polymorphism of* CNR2*, which encodes CB2R, is related to schizophrenia [31, 32], depression [22], and bipolar disorder [33]. The deletion of CB2Rs in mice also induces schizophrenia-like symptoms, such as impairment in sensory-motor gating and an increase in depressive behavior [34]. In addition, CB2R KO mice display a deficit in long-term memory assessed in a step-down passive avoidance test [27, 34], which probes the functions of the hippocampus, entorhinal cortex, parietal cortex, and/or amygdala (for review, see [35]). However, it has not been determined whether CB2R KO mice also display other phenotypes resembling schizophrenia-related behaviors. Patients with schizophrenia have working memory deficits (for review, see [36]) and impaired functions of the hippocampus (for reviews, see [37, 38]) and amygdala (for reviews, see [39, 40]). These features are often recapitulated in animal models of schizophrenia (for reviews, see [41, 42]). Because CB2R is implicated in schizophrenia in humans and schizophrenia-associated behaviors in mice, we hypothesized that CB2R KO mice might have deficits in working memory and long-term memory dependent on the hippocampus and/or amygdala.

Here, we tested spatial working memory and long-term fear memory of CB2R KO mice. Our data indicated that contextual fear memory, which is dependent on the hippocampus, was impaired in CB2R KO mice whereas hippocampus-independent, cued fear memory was not affected. In addition, spatial working memory was enhanced in CB2R KO mice. However, acute blockade of CB2Rs by AM630 did not alter memory, motor activity, or anxiety. These results suggest that the roles of CB2Rs in memory are diverse depending on memory types and/or brain areas.

## 2. Materials and Methods

### 2.1. Animals

CB2R KO mice (The Jackson Laboratory, Bar Harbor, ME; Stock number 005786) were originally created by Deltagen (San Mateo, CA) on the background of C57BL/6J mice. We crossed homozygous KO mice (CB2R^−/−^) with C57BL/6J mice (The Jackson Laboratory) to obtain heterozygous CB2R^+/−^ mice. CB2R^+/−^ mice were bred with each other to generate littermates of CB2R^+/+^ and CB2R^−/−^. These wild type (WT) and KO mice of either sex were used for experiments at age 2.5–4 months. Male C57BL/6J mice at age 2.5–3 months were also used for AM630 administration (Figures 5 and 6). Animals were group-housed (3-4 mice per cage) in a temperature- and light-controlled room (23°C and the light/dark cycle of 6 AM/6 PM) with free access to food and water. Before experiments, the experimenter handled mice daily for 5 days, 5 min a day. On the day of experiment, mice were placed in the test room >1 h before tests. All experiments were conducted in accordance with the animal use protocol that was approved by the Institutional Animal Care and Use Committee of Georgia Regents University. We genotyped mice using REDExtract-N-Amp Tissue PCR Kit (Sigma-Aldrich, St. Louis, MO) and the following primers: GGGGATCGATCCGTCCTGTAAGTCT, GACTAGAGCTTTGTAGGTAGGCGGG, and GGAGTTCAACCCCATGAAGGAGTAC.

### 2.2. Drug Administration

Male C57BL/6J mice (2.5–3 months old) were injected with AM630 (Tocris, Minneapolis, MN), a specific CB2R antagonist, at 3 mg/kg (i.p., ~250 *μ*L per mouse). Age- and sex-matched control mice were administered with vehicle (0.9% NaCl solution with 5% DMSO and 5% Tween 80). An AM630 stock solution was made in DMSO and diluted immediately before injection in a NaCl-Tween 80 solution. In the fear conditioning experiments, mice were treated with AM630 or vehicle 3 min after the conditioning session. In the experiments of Y-maze, open field arena, and elevated zero maze, another group of animals (i.e., drug-naive) were administered with AM630 or vehicle 1 h before the behavioral tests.

### 2.3. Fear Conditioning

The fear conditioning chamber (18 × 18 × 28.5 cm^3^; Coulbourn Instruments, Whitehall, PA) was made of metal on two sides and transparent plastic on the other sides with an opening at the upper part of the walls. The floor of the chamber consisted of a stainless steel grid, to which an electric shock was applied. The conditioning chamber was housed in a soundproof isolation cubicle (Coulbourn Instruments) and illuminated with 7-lux white light from a lamp attached to a wall of the chamber. Auditory tones for fear conditioning were generated by a sound generator (Sony Audio Control Center STR-DH130) and delivered to a speaker attached to the conditioning chamber. For fear conditioning, a mouse was placed in the chamber for 90 s and then presented with a tone (80 dB) for 30 s. During the last 2 s of the tone, an electric shock (0.5 mA, 2 s) was delivered to the grid floor. The behavior of mice was monitored and recorded by a camera mounted on the ceiling of the chamber and analyzed by the FreezeFrame 4 software (Actimetrics, Wilmette, IL). When mice showed no noticeable movement for ≥1 s, it was counted as freezing behavior. The 2 min session of a 90 s rest and a 30 s tone was repeated 3 times continuously for a given mouse. Immediately after the conditioning, mice were returned to home cages. The conditioning chamber was wiped with 70% ethanol after testing each animal.

For contextual fear memory tests, mice were placed, 24 h after the conditioning, in the same conditioning chamber for 5 min and the freezing behavior was monitored. Cued fear memory was tested 1-2 h after the contextual memory test with visual and odorous modifications of the conditioning chamber. Acetic acid (5%) in a Petri dish was placed in the isolation cubicle, but outside the chamber, for a new odor. Visual context was altered by lining the walls and floor of the chamber with paper and/or placing an opaque plastic bucket (16.5 cm diameter and 12.5 cm height) in the chamber. For additional visual modifications, the color of light in the chamber was changed to yellow. A mouse was put in the chamber for 2 min and then presented with a tone (80 dB) for 3 min. Animals were returned to home cages 1 min after the termination of the tone. During the cued fear memory test, two WT mice escaped from the chamber through the upper opening and thus were removed from the analysis.

### 2.4. Y-Maze

A continuous spontaneous alternation test was performed in a Y-maze (San Diego Instruments, San Diego, CA). The Y-maze consisted of three arms at 120° and was made of beige plastic. Each arm was 7.5 cm wide and 38 cm long, and its three sides (except for the side adjoining the other arms) were surrounded by 12.5-cm high walls. The floor of the Y-maze was covered with a sawdust bedding material. Between each trial, the sawdust was mixed and redispersed to remove or randomize odor trails. Distal visual cues were placed around the Y-maze. A mouse was placed in the Y-maze and allowed to explore for 3 min under the illumination of 100 lux. Mouse behavior was monitored, recorded, and analyzed by a webcam (C920, Logitech, Newark, CA) and the Any-Maze software (Stoelting, Wood Dale, IL). A mouse was considered to have entered an arm if the whole body (except for the tail) entered the arm and to have exited if the whole body (except for the tail) exited the arm. If an animal consecutively entered three different arms, it was counted as an alternating triad. Because the maximum number of triads is the total number of arm entries minus 2, the score of alternation was calculated as “the number of alternating triads/(the total number of arm entries − 2).”

### 2.5. Open Field Arena

Exploratory motor activity and anxiety were tested in an open field arena (40 × 40 cm^2^ with 30-cm high walls), which was made of wood coated with black plastic. The floor was covered with a sawdust bedding material. After each trial, the sawdust was redispersed to remove odor trails. A mouse was placed in the open field arena and allowed to explore for 5 min under 100-lux illumination. Mouse behavior was monitored, analyzed, and recorded by a webcam (Logitech C920) and the Any-Maze software (Stoelting). The area of 20 × 20 cm^2^ in the middle of the arena was set as a center area in the analysis software. The tendency of a mouse to avoid this center area was used as an indication of anxiety level.

### 2.6. Elevated Zero Maze

Anxiety levels of mice were also tested in an elevated zero maze (San Diego Instruments). The zero maze was a continuous circular track made of beige plastic. The width of the track was 5 cm and the inner diameter was 48 cm. Two opposite quadrants of the circular track (referred to as “closed quadrants”) were surrounded by two 15-cm high walls along the edge of the track. The edges of the other two quadrants of the track (referred to as “open quadrants”) were lined with 1-cm high rails. The zero maze was elevated 51 cm above the floor by steel legs. After each trial, the maze was wiped with 70% ethanol. A mouse was placed on the zero maze and allowed to explore for 5 min under 200-lux illumination. Mouse behavior was monitored, recorded, and analyzed by a webcam (Logitech) and the Any-Maze software (Stoelting). A mouse was considered to have entered or exited a closed quadrant if the whole body (except for the tail) entered or exited, respectively, the quadrant.

### 2.7. Statistics

Comparisons between two groups were made with Student's *t*-tests with a two-tailed confidence level of *P* < 0.05.

## 3. Results

### 3.1. Contextual, but Not Cued, Fear Memory Is Impaired in CB2R KO Mice

**Figure 1:**
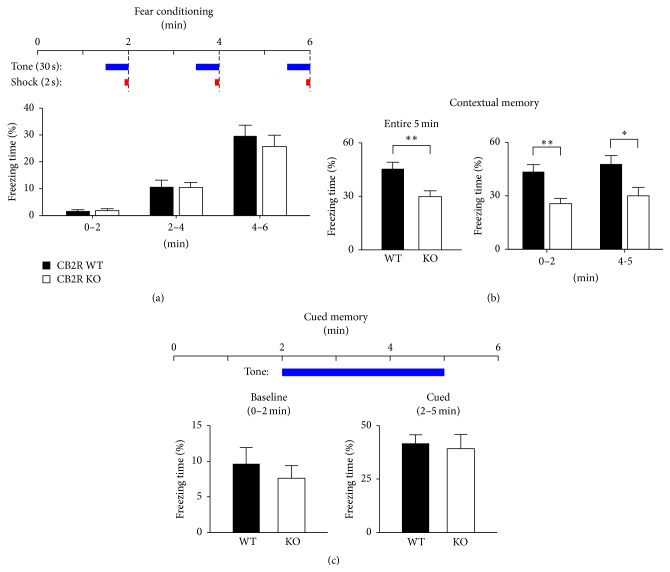
Contextual, but not cued, fear memory of CB2R KO mice is impaired. (a) Baseline freezing behavior of mice during the conditioning period. A mouse in a fear conditioning chamber was presented with a tone for 30 s, 3 times every 2 min. During the last 2 s of the tone, an electric foot shock was delivered. The freezing time of KO mice was similar to that of WT mice in each of the 2 min periods. (b) Contextual fear memory was tested 24 h after the fear conditioning. Mice were placed in the same conditioning chamber for 5 min and the freezing time was measured. The freezing behavior was also analyzed in the early (0–2 min) and late (4-5 min) phases of the 5 min period. ^*∗∗*^ *P* < 0.01; ^*∗*^ *P* = 0.02; *t*-test. (c) Cued fear memory was assayed after the contextual memory test in a modified conditioning chamber. A tone was presented for 3 min and the freezing behavior was monitored before (for 2 min) and during the tone. Error bars represent SEM.

For fear conditioning, CB2R WT and KO mice were presented with a 30 s tone and a 2 s electric foot shock, 3 times every 2 min (Figure 1(a)). For the first 2 min, WT and KO mice spent 1.6 ± 0.5% (*n* = 18) and 1.7 ± 0.8% (*n* = 11), respectively, of the time being frozen (*P* = 0.86, *t*-test; Figure 1(a)), suggesting that the two groups of mice had similar baseline freezing behavior. During the second 2 min period, the freezing time increased to about 10% for both WT and KO mice, and there was again no difference between the two strains (*P* = 0.95, *t*-test; Figure 1(a)). For the last 2 min of the conditioning session, the freezing time of KO mice (26 ± 4%) was not significantly different from that of WT mice (30 ± 4%) (*P* = 0.52, *t*-test; Figure 1(a)). This result implies that WT and KO mice had similar baseline responses to electric foot shocks.

On the next day, the same animals were tested for long-term, contextual fear memory by being placed in the same conditioning chamber for 5 min without a tone or a foot shock. During the 5 min exposure to the same context, WT mice showed freezing behavior for 45 ± 4% of the time, whereas KO mice froze for a significantly shorter period of time, 30 ± 3% (*P* = 0.0078, *t*-test; Figure 1(b)). This result suggests that the contextual fear memory might be impaired in CB2R KO mice. However, an alternative interpretation could be that the KO mice quickly noticed after being placed in the chamber that a foot shock was not delivered and thus disconnected the context from fear. To test for this possibility, we analyzed the freezing behavior separately during the early (0–2 min) and late (4-5 min) periods of the 5 min exposure. For the first 2 min, the freezing time of the KO mice (26 ± 3%) was still significantly shorter than that of the WT mice (44 ± 4%) (*P* = 0.0027, *t*-test; Figure 1(b)). Similarly, the KO mice displayed significantly shorter freezing time (30 ± 5%) during the last 1 min period than the WT mice did (48 ± 5%) (*P* = 0.020, *t*-test; Figure 1(b)). This result implies that the mice did not adapt to a shock-free environment but showed consistent freezing behavior throughout the 5 min exposure period. Together, our data support the idea that the long-term contextual fear memory, which is dependent on the hippocampus, was impaired in CB2R KO mice.

Next, we assayed hippocampus-independent, cued fear memory [50–53]. Mice were placed in the conditioning chamber with modified visual and odorous cues for 2 min without a shock or a tone and then for 3 min with a tone. For the first 2 min, WT and KO mice showed no difference in baseline freezing behavior in the new context: 10 ± 2% and 7.6 ± 2% freezing time for WT (*n* = 16) and KO (*n* = 11), respectively (*P* = 0.52, *t*-test; Figure 1(c)). When presented with a tone for 3 min, the mice displayed more freezing, but the freezing time of KO mice (39 ± 7%) was again not significantly different from that of WT mice (42 ± 4%) (*P* = 0.74, *t*-test; Figure 1(c)). These results suggest that the impairment of long-term fear memory in CB2R KO mice was specific for hippocampus-dependent processes.

### 3.2. Spatial Working Memory Is Enhanced in CB2R KO Mice

**Figure 2:**
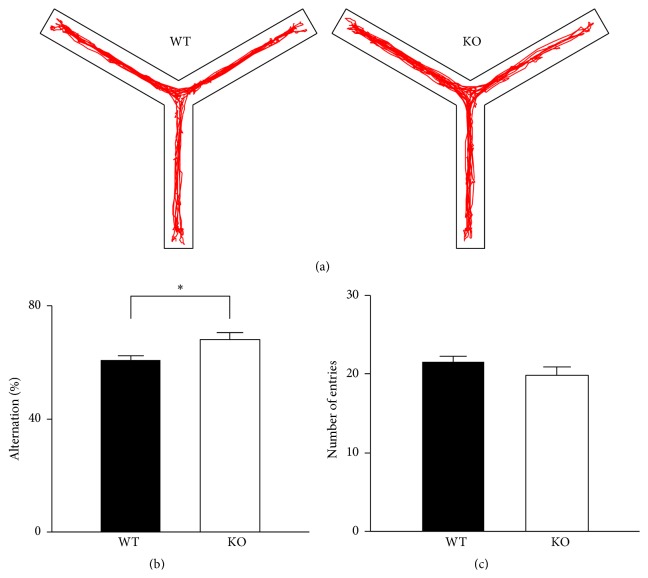
Spatial working memory of CB2R KO mice is enhanced when assessed with a spontaneous alternation test in a Y-maze. (a) Representative tracking of mouse movement for 3 min in a Y-maze. Consecutive entries into three different arms were counted as an alternation. (b) KO mice displayed a higher rate of spontaneous alternation compared with WT mice. ^*∗*^ *P* = 0.012, *t*-test. (c) The total number of arm entries was not significantly different between the two strains. Error bars represent SEM.

We performed a spontaneous alternation test in a Y-maze to assess spatial working memory (Figure 2(a)). Alternation of arm entries is driven by an instinct of a mouse to visit a novel place and requires the mouse to remember which arms it entered in its immediately previous exploration (for review, see [54]). During the 3 min test session in a Y-maze, CB2R KO mice had a significantly higher probability of alternating three consecutive entries (68 ± 2%; *n* = 12) than WT mice did (61 ± 2%; *n* = 19) (*P* = 0.012, *t*-test; Figure 2(b)). This result suggests that the spatial working memory of CB2R KO mice was enhanced compared with that of WT mice. Alternatively, it is also possible that CB2R KO mice had higher exploratory motivation and thus stayed in each arm for a shorter period of time, resulting in a less temporal burden for memory storage. To test for this possibility, we analyzed the total number of arm entries for 3 min, but both WT and KO mice showed similar number of entries (*P* = 0.19, *t*-test; Figure 2(c)). Together, these data imply that CB2R KO had enhanced spatial working memory.

### 3.3. Motor Activity and Anxiety in an Open Field Arena Are Not Affected by KO of CB2Rs

**Figure 3:**
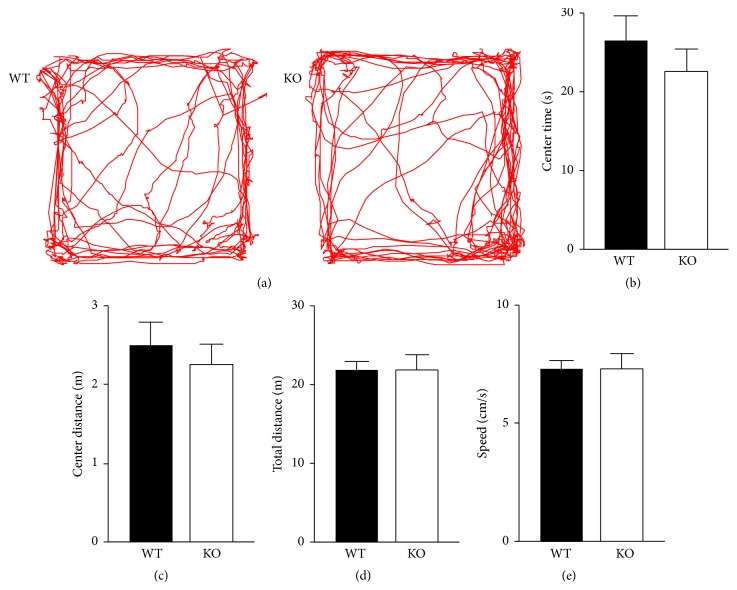
The levels of motor activity and anxiety assessed in an open field arena are normal in CB2R KO mice. (a) Representative tracking of mouse movement for 5 min in an open field arena (40 × 40 cm^2^). (b–e) During the 5 min exploration period, KO and WT mice showed no significant difference from each other in the time spent in the center area (20 × 20 cm^2^) of the arena (b), the travel distance in the center area (c), the total travel distance in the arena (d), or the mean speed of movement (e). Error bars represent SEM.

Fear memory tests and a Y-maze test involve motor activity and/or anxiety of mice. Although the analyses of these tests (Figures 1 and 2) implied that the mobility and basal freezing behavior of CB2R KO mice were similar to those of WT mice, we further examined these properties of mice in an independent assay. An open field arena was used to assess both motor activity and anxiety while mice explored the arena for 5 min (Figure 3(a)). As an assay of anxiety in an open field arena, the exploration time and travel distance in the center area of the arena were analyzed. CB2R KO mice spent 23 ± 3 s (*n* = 11) in the center area and it was not significantly different from the time spent by WT mice (27 ± 3 s; *n* = 18) (*P* = 0.40, *t*-test; Figure 3(b)). The distance traveled in the center area by KO mice (2.3 ± 0.3 m) was also similar to that by WT mice (2.5 ± 0.3 m) (*P* = 0.58, *t*-test; Figure 3(c)). These results suggest that there was no difference in anxiety levels between the two strains of mice. The total distance traveled by the KO mice for 5 min (21.8 ± 1.9 m) was not different from that by the WT mice (21.8 ± 1.1 m) (*P* = 0.997, *t*-test; Figure 3(c)). The mean speed of travel was 7.3 ± 0.4 cm/s for WT mice and 7.3 ± 0.7 cm/s for KO mice (*P* = 0.98, *t*-test; Figure 3(e)). These results indicate that the CB2R deletion did not affect motor activity.

### 3.4. CB2R KO and WT Mice Have Similar Levels of Anxiety in an Elevated Zero Maze

**Figure 4:**
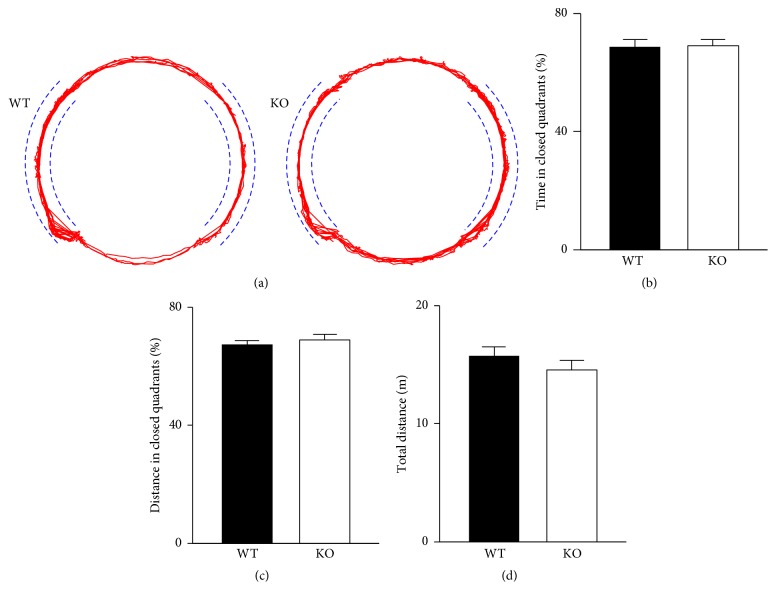
The anxiety level of CB2R KO mice is similar to that of WT mice when assayed in an elevated zero maze. (a) Representative tracking of mice in an elevated zero maze for 5 min. Dashed lines indicate the walls in two closed quadrants. (b-c) KO and WT mice spent similar time (b) and traveled similar distances (c) in the closed quadrants of the zero maze. (d) The total travel distance of KO mice in the zero maze for 5 min was not significantly different from that of WT mice. Error bars represent SEM.

The anxiety of CB2R KO and WT mice was additionally assessed in an elevated zero maze, which consisted of two quadrants with walls and two other quadrants without walls (Figure 4(a)). The more anxious a mouse is, the more time it will spend in walled, or closed, quadrants. During a 5 min test session, CB2R WT (*n* = 18) and KO (*n* = 11) mice spent the same amount of time (69 ± 2% of the total time) in the closed quadrants (*P* = 0.89, *t*-test; Figure 4(b)). The distance traveled in the closed quadrants was 67 ± 1% and 69 ± 2% of the total travel distance for WT and KO mice, respectively (*P* = 0.54, *t*-test; Figure 4(c)). The two mouse groups also displayed similar levels of overall motor activity in an elevated maze because the total travel distance was 15.7 ± 0.8 m for WT and 14.5 ± 0.8 m for KO mice (*P* = 0.33, *t*-test; Figure 4(d)). This result supports the idea that the anxiety levels of CB2R KO mice were similar to those of WT mice.

### 3.5. Acute Blockade of CB2Rs Has Little Effect on Memory

**Figure 5:**
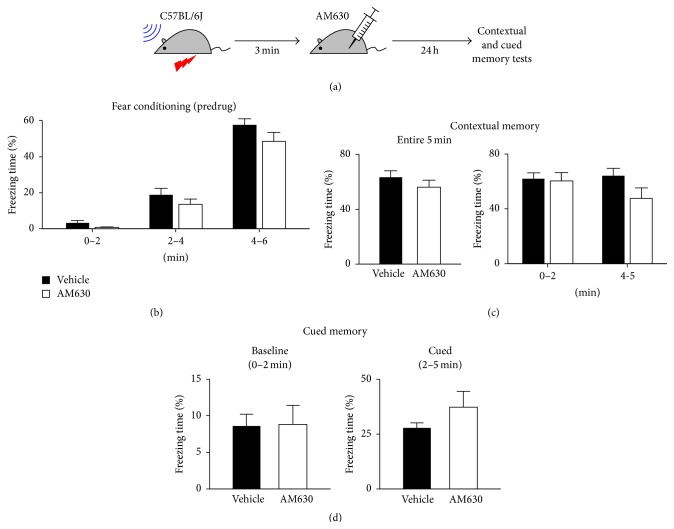
Acute blockade of CB2Rs had little effect on fear memory. (a) Adult male C57BL/6J mice were fear-conditioned with a tone and a foot shock, as illustrated in Figure 1(a). The animals were injected with AM630 (3 mg/kg, i.p.), a CB2R antagonist, 3 min after the conditioning. (b) Baseline freezing behavior of mice during the conditioning session. The mice were randomly divided into two groups for injection with AM630 or vehicle. (c) Contextual fear memory of AM630-treated mice was not significantly different from that of control mice. The freezing time was counted during a 5 min session and also analyzed for the first 2 and the last 1 min of the session. (d) Cued fear memory was not affected by acute treatment with AM630. A tone was presented for 3 min. There was no significant difference in freezing behavior between the two groups either before or during the tone. Error bars represent SEM.

Next, we tested whether acute blockade of CB2Rs also induced similar effects to those of chronic KO of CB2Rs. C57BL/6J mice were fear-conditioned as described in Figure 1(a) and then randomly divided into two groups with 11 mice per group. These two groups were not different from each other in freezing behavior during the 6 min conditioning session (*P* > 0.1 in each 2 min period; Figures 5(a) and 5(b)). Mice in the test group were injected with AM630 (3 mg/kg; i.p.), a CB2R antagonist, 3 min after the conditioning and control mice were administered with vehicle. In the contextual memory test on the next day, AM630-treated mice froze 56 ± 5% of the 5 min test period and this value was not significantly different from that of vehicle-treated mice (64 ± 5%; *P* = 0.30, *t*-test; Figure 5(c)). When we analyzed the freezing behavior for the early 2 min (*P* = 0.84, *t*-test; Figure 5(c)) and the last 1 min (*P* = 0.094, *t*-test; Figure 5(c)), the two groups still did not differ from each other. In the cued memory test, the freezing time of AM630-treated mice (37 ± 7%) was not significantly different from that of control mice (28 ± 2%; *P* = 0.23, *t*-test; Figure 5(d)). Their basal freezing before the tone was also similar to each other (*P* = 0.94, *t*-test; Figure 5(d)). These data indicate that acute blockade of CB2Rs altered neither contextual nor cued fear memory.

**Figure 6:**
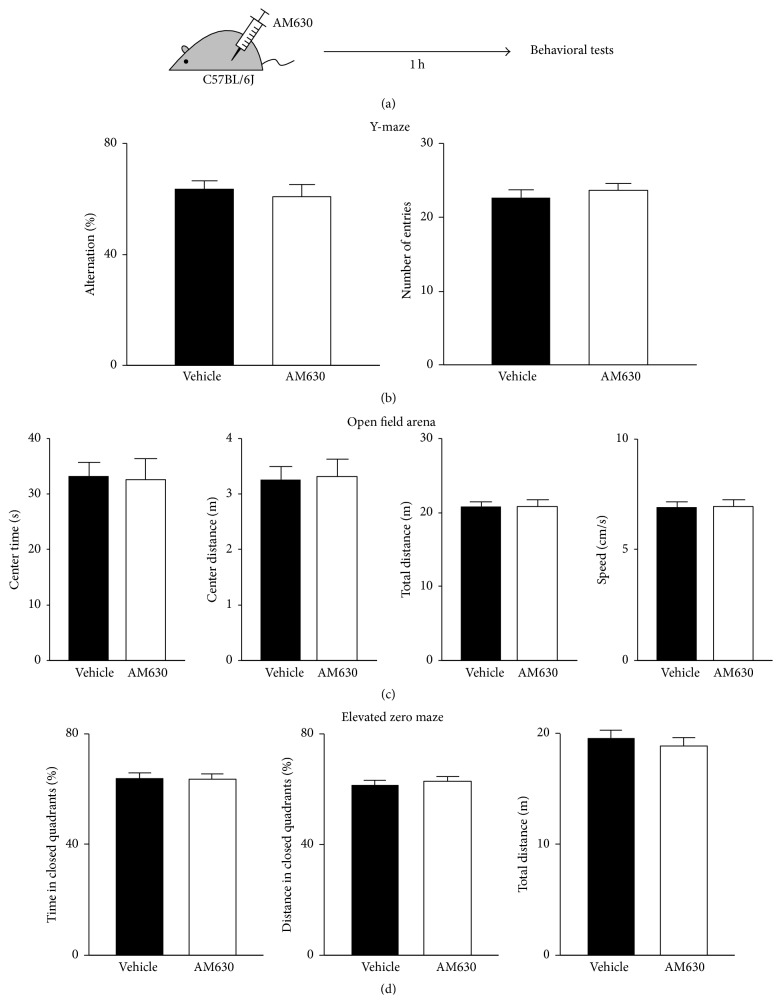
Acute blockade of CB2Rs had no effect on working memory, locomotion, or anxiety. (a) Adult male C57BL/6J mice were injected with AM630 (3 mg/kg, i.p.) and, 1 h after the injection, used for behavioral tests in a Y-maze, an open field arena, and an elevated zero maze. (b) The rate of spontaneous alternation or the number of arm entries of AM630-treated mice in a Y-maze was not different from that of control mice. (c) In an open field arena, AM630- and vehicle-injected mice displayed similar behavioral patterns in the time spent in the center area, distance traveled in the center area, total travel distance, and travel speed. (d) In an elevated zero maze, the time and distance in the closed quadrants and the total travel distance of AM630-administered mice were similar to those of control mice. Error bars represent SEM.

We examined working memory, exploratory behavior, and anxiety using another group of drug-naive mice. C57BL/6J mice were injected with AM630 (3 mg/kg; i.p.; *n* = 12) or vehicle (*n* = 12) and used 1 h later for behavioral tests (Figure 6(a)). In the working memory test in a Y-maze, AM630-treated mice showed a 61 ± 5% rate of spontaneous alternation and it was similar to that of vehicle-injected mice (64 ± 3%; *P* = 0.60, *t*-test; Figure 6(b)), suggesting that spatial working memory was not affected by acute administration of AM630. The number of arm entries was also similar in the two groups (*P* = 0.50, *t*-test; Figure 6(b)). In the assay of open field arena, four parameters were analyzed—the time spent in the center area, distance traveled in the center area, total travel distance, and travel speed—but none of them was altered by AM630 (*P* > 0.8, *t*-tests; Figure 6(c)). The behavior of AM630-treated mice on an elevated zero maze was not significantly different from that of control animals when the time and distance in closed quadrants and the total travel distance were analyzed (*P* > 0.5, *t*-test; Figure 6(d)). The data from open field arena and zero maze experiments imply that the acute treatment with AM630 had little effect on locomotor activity and anxiety.

## 4. Discussion

The present study shows that CB2R KO mice have, compared with WT mice, enhanced spatial working memory, impaired contextual fear memory, and normal cued fear memory (Table 1). These changes in memory are not caused by confounding effects of alterations in motor activity or anxiety because CB2R KO and WT mice displayed similar behavioral phenotypes in an open field arena and an elevated zero maze. Given that the hippocampus is involved in contextual, not cued, fear memory [50–53], our results imply that the effects of CB2R deletion on memory are variable depending on memory types and/or brain areas. Our data also indicate that acute blockade of CB2Rs by AM630 has no effect on memory, motor activity, or anxiety of mice, implying that the downregulation of CB2Rs might need to be prolonged to induce such effects.

CB2Rs have been implicated in the regulation of synaptic and neuronal functions. In the hippocampus, excitatory synaptic transmission is increased by chronic activation of CB2Rs [28] and dendritic spine density is reduced by deletion of CB2Rs [27]. Acute stimulation of CB2Rs decreases the amplitude of spontaneous inhibitory synaptic transmission in the entorhinal cortex [29] and inhibits potassium-evoked GABA release from synaptosomes [30], but not in the hippocampus [28]. Chronic activation of CB2Rs increases GABA_A_ receptor expression [25], although it does not change inhibitory synaptic transmission in the hippocampus [28]. CB2R agonists increase chloride conductance and reduce membrane excitability of cortical [26], not hippocampal [28], neurons. These studies suggest that the cellular effects of CB2R activation appear to be diverse depending on brain areas. Taken together with our current results, the spatial specificity of CB2R functions might be an important factor in determining the role of CB2Rs in modulating synaptic transmission and memory. For a complete understanding of the cellular mechanisms of CB2R effects, it will be necessary to determine the roles of CB2Rs in regulating diverse properties of synaptic transmission, for example, presynaptic release properties, postsynaptic responsiveness, short-term plasticity, and long-term plasticity.

Our observation of impaired long-term memory is in accord with the previous report of deficits in step-down passive avoidance memory in CB2R KO mice [27, 34]. The passive avoidance memory depends on functions of the hippocampus, entorhinal cortex, parietal cortex, and/or amygdala [35]. On the other hand, the fear conditioning tests probe hippocampus-dependent and hippocampus-independent memory separately [50–53]. Our data revealed that a deficit in long-term fear memory is specific for hippocampus-dependent, contextual memory. In the open field test and elevated zero maze test, CB2R KO mice were not different from WT mice in terms of exploratory activity and anxiety (Figures 3 and 4). However, it was previously reported that CB2R KO mice traveled a significantly shorter distance in an open field arena and spent less time in the open arms of an elevated plus maze compared with WT mice [34]. It is unclear what caused the difference between our results and the previous report, but one of the reasons could be the difference in mouse background. We obtained C57BL/6J-based KO mice and bred them with C57BL/6J mice, whereas, in the other study, C57BL/6J-based KO mice were crossed with CD1 mice [34]. Given that some effects of cannabinoid receptor KO are variable depending on the strain background [43, 55, 56], the mouse background might have contributed to the discrepancy between the two studies. One of the advantages of using our mouse strain is that potential confounding effects resulting from changes in motor activity and/or anxiety can be ruled out. In the CB2R KO mice that we used, amino acids 26–137 of CB2R (total 347 amino acids) were deleted. However, a different region was removed [57] in the CB2R KO mice used in the other study [34]. If the sequence upstream of the deleted region is translated, a peptide with potential biological activity could be generated. In addition, it is possible that the sequence downstream of the deleted region could be translated if the neocassette (hence the stop codon in the cassette) is spliced out. Because different regions are missing in the two strains of CB2R KO mice, such partial translation, if any, would produce peptides that might possess distinct function in each mouse strain, resulting in differences in phenotypes. Whether fragments of CB2Rs are expressed in KO mice remains to be determined.

We used both male and female mice but the previous study [34] included only males. However, the sex difference might not account for the difference in the results because our conclusion remained consistent even when we analyzed only male mice in our data set. With only males, the freezing time of KO mice (*n* = 8) in the contextual memory test was shorter than that of WT mice (*n* = 13; *P* = 0.038, *t*-test), and there was no difference in the cued memory test between WT (*n* = 12) and KO (*n* = 8) mice (*P* = 0.91, *t*-test). The probability of alternation in the Y-maze test with male KO mice (*n* = 8) was higher than that with male WT mice (*n* = 13; *P* = 0.024, *t*-test). There was no difference between the two male groups in any analysis of the open field test and zero maze test (*P* > 0.2, *t*-tests).

As introduced earlier, the abnormality of* CNR2* in humans is related to schizophrenia [31, 32] and CB2R KO mice display schizophrenia-like phenotypes, for example, impairment in sensory-motor gating and an increase in depressive behavior [34]. Individuals with schizophrenia have deficits in working memory (for review, see [36]), hippocampal functions (for reviews, see [37, 38]), amygdala functions (for reviews, see [39, 40]), and anxiety (for review, see [58]). However, our study indicates that CB2R KO mice display improved working memory, normal cued fear memory (which requires amygdala function), and normal anxiety levels. Although CB2R KO mice clearly recapitulate some of the schizophrenia-related phenotypes, our data imply that the degree of the recapitulation needs to be scrutinized.

Acute administration of the CB2R antagonist AM630 in vivo had little effects on memory, locomotion, and anxiety in our experiments. At the cellular level, we have observed that treatment of hippocampal slice cultures with SR144528, a CB2R antagonist, even for 7–10 d did not affect excitatory synaptic transmission [28]. Therefore, it appears that the downregulation of CB2Rs needs to last long to produce any effect on synaptic functions and memory. Acute intraperitoneal administration of AM630 at 3 mg/kg, which we also used, into mice has been reported to impair long-term memory in a passive avoidance test [27] and increase anxiety in a light-dark box test [25]. In contrast, the same drug at the same dose in our experiments did not produce any significant effect on long-term fear memory or anxiety. The reason for this discrepancy is unclear but the differences in mouse strain and/or the types of behavioral assays might have contributed to the unmatched results. Although both CB2R WT mice and C57BL/6J mice are considered control groups, the former (Figure 1(a)) displayed less freezing response to a foot shock than the latter (Figure 5(b)). C57BL/6J is a congenic background of CB2R WT mice, but their genetic compositions might not be identical to each other, possibly resulting in differences in phenotypes [59]. Furthermore, context- or cue-induced freezing behavior of CB2R WT mice (Figures 1(b) and 1(c)) is not the same as that of vehicle-injected C57BL/6J mice (Figures 5(c) and 5(d)). In addition to the genetic mismatch, the stress caused by vehicle injection into C57BL/6J mice and/or vehicle itself might be another factor contributing to the phenotypic differences between the two control groups. To avoid these confounding effects, comparisons were made only between CB2R WT and KO mice or between C57BL/6J groups.

One of the possible mechanisms for the delayed effects of CB2R downregulation could be its effects on neurogenesis. Activation of CB2Rs promotes the proliferation of neural progenitor cells in vivo in the hippocampus [60, 61] and subventricular zone [62], as well as neural stem cells in culture [63]. CB2R KO mice display a decrease in neurogenesis in the hippocampus [60]. Interestingly, blockade of hippocampal neurogenesis in adult mice impairs contextual fear memory but not cued fear memory or spatial memory [64]. It would be an important task to determine whether the effect of CB2R KO on contextual memory is mediated by the disruption of hippocampal neurogenesis. CB2R KO mice have impairment in eye-specific segregation of retinal projections to the dorsal lateral geniculate nucleus [65] and an increase in retinal sensitivity [66]. Therefore, if the vision of CB2R KO mice is abnormal, it might influence the contextual learning because this form of learning requires recognition of visual context. If vision is one of the factors contributing to the deficit in contextual memory in CB2R KO mice, it would still remain puzzling how abnormal vision, if any, does not interfere with, but rather enhanced, spatial working memory, of which the acquisition and recall also require visual cues.

Although CB2Rs are expressed in the brain, the expression level of CB2Rs in the peripheral immune system is much higher than that in the central nervous system (for reviews, see [4, 67]). Therefore, the deletion of CB2Rs in the immune system, not only in the brain, should be taken into account when data from CB2R KO mice are interpreted. The immune system can enhance the ability of learning and memory under quiescent conditions (i.e., without inflammation or injury) via interactions among T cells, microglia, and neurons, whereas a surge of cytokines under inflammatory conditions can impair the processes of learning and memory (for review, see [68]). In relation to immune functions, CB2R KO mice are more vulnerable to experimental autoimmune encephalomyelitis, allergic dermatitis, and bacterial infection (for review, see [69]). It needs to be determined in the future whether the compromised immune functions in CB2R KO mice affect the processes involved in learning and memory.

CB1R KO mice display impaired extinction, but normal acquisition, of both spatial reference memory [44, 45] and cued fear memory [48]. Working memory is also reduced in CB1R KO mice [43]. Contextual fear memory of CB1R KO mice was reported to be impaired [46] or enhanced [47], whereas passive avoidance memory was unaffected [49]. Combined with our data (Table 1), these results indicate that the normal acquisition of cued fear memory is common for both CB1R KO and CB2R KO mice, but the changes in working memory are opposite in CB1R KO and CB2R KO mice. Acute administration of a CB1R agonist into rodents impairs spatial reference memory [70–72], working memory [71, 73], and contextual fear memory [74]. In contrast, acute treatment of mice with a CB2R agonist enhances passive avoidance memory whereas a CB2R antagonist impairs it [25] but not fear memory (Figure 5). Taken together, these studies reveal that CB1Rs and CB2Rs have both similar and distinct roles in modulating memory. Given that Δ^9^-THC, the major psychoactive component of cannabis, and endocannabinoids (e.g., anandamide and 2-arachidonoylglycerol) can activate both CB1Rs and CB2Rs [16–18], it will be an important task to determine how various effects of Δ^9^-THC on cognitive functions are mediated by each type of cannabinoid receptors. Once the neurocognitive effects of each receptor are fully characterized, CB1R or CB2R can be selectively targeted for pharmacological therapeutics to induce only desired effects while avoiding unwanted ones.

## Figures and Tables

**Table 1:** Changes in memory of cannabinoid receptor KO mice. Changes in various types of memory of CB1R KO and CB2R KO mice, compared with WT mice, are summarized from our current results and other studies. Spatial working memory was assayed in a spontaneous alternation test in a Y-maze. Spatial reference memory was measured in a Morris water maze. ↑: enhanced; ↓: impaired; =: unaffected.

	CB2R KO		CB1R KO	
Spatial				
Working	↑	Figure 2(b)	↓	[43]
Reference			Acquisition = Extinction ↓	[44, 45]
Conditioned fear				
Contextual	↓	Figure 1(b)	↓ ↑	[46] [47]
Cued	=	Figure 1(c)	Acquisition = Extinction ↓	[48]
Passive avoidance	↓	[27, 34]	=	[49]
